# Current Concepts in Imaging Diagnosis and Screening of Blunt Cerebrovascular Injuries

**DOI:** 10.3390/tomography8010033

**Published:** 2022-02-07

**Authors:** Tiffany Y. So, Apurva Sawhney, Lei Wang, Yi Xiang J. Wang

**Affiliations:** Department of Imaging and Interventional Radiology, The Chinese University of Hong Kong, Prince of Wales Hospital, Hong Kong SAR, China; asawhney@cuhk.edu.hk (A.S.); leiwang001@link.cuhk.edu.hk (L.W.); yixiang_wang@cuhk.edu.hk (Y.X.J.W.)

**Keywords:** blunt cerebrovascular injury, neck injuries, vertebral arteries, carotid arteries, stroke

## Abstract

Blunt cerebrovascular injury (BCVI) is an often underrecognized injury occurring in the carotid or vertebral arteries, associated with a risk of ischemic stroke and potential for poor neurological outcome or death. Computed tomographic angiography (CTA) is the most common modality for initial screening and diagnosis. Vessel wall intimal injuries, intraluminal thrombus, dissection, intramural hematoma, pseudoaneurysm, vessel transection, and arteriovenous fistula, are potential findings to be considered in approach to imaging. Identification of high-risk trauma patients based on clinical and radiological risk factors can determine patients at risk of BCVI for targeted screening.

## 1. Introduction

Blunt cerebrovascular injury (BCVI) is a potentially devastating, yet often underrecognized, non-penetrating injury which occurs in the carotid or vertebral arteries. Most often associated with high energy trauma, BCVI most commonly occurs as a complication of high-speed motor vehicle accidents, pedestrian versus car accidents, falls from heights or assaults [1,2,3]. Other mechanisms of injury include direct head or neck injuries resulting from assaults or suicide attempts by hanging [4]. Reported incidences are in the range of 0.1–3% of all trauma admissions [2,5,6,7,8]. However, true incidences may be much higher, as BCVI is often masked by the presence of other cranial, facial or cervical injuries and variable neurologic deficits, which can lead to missed diagnoses [1,9]. Recent data shows that ischemic stroke is the main cause of morbidity and mortality from BCVI, which is linked to underdiagnosis or delayed diagnosis. In untreated carotid injuries, morbidity rates of 32–67% and mortality rates of 17–38% have been reported. Morbidity rates of 14–24% and mortality rates of 8–18% have also been reported for untreated vertebral injuries [10]. Screening of high-risk trauma patients may facilitate earlier diagnosis and decision of appropriate treatment. This article discusses the imaging evaluation techniques and common imaging findings of BCVI, and reviews the current concepts in BCVI screening.

## 2. Imaging Techniques

Computed tomographic angiography (CTA) is the preferred choice for initial screening and diagnosis of BCVI, showing considerable equivalence to 4-vessel digital subtraction angiography (DSA) in diagnostic accuracy [11]. With technological advancements in CT throughout the years and widespread availability of multidetector scanners, resolution of CTA has significantly improved. Most guidelines recommend the use of ≥16 section multidetector CTA for BCVI detection; these showing sensitivities of 64–98% and specificities of 92–100% compared to DSA for BCVI diagnosis in recent studies [12,13,14,15,16,17,18,19,20]. Variations in CTA protocol or radiologist interpretation, artifacts such as dental or motion artifact, or incorrect contrast timing during CTA are factors which may reduce the accuracy of CTA and contribute to a range of reported CTA sensitivity estimates. Nevertheless, studies suggest that CTA can detect the majority of clinically significant injuries, with most missed injuries being low-grade with low incidences of subsequent ischemic stroke [16]. In critically injured patients, CTA is readily accessible and can be performed in minutes. This is a definite advantage over DSA, which requires transportation to the angiography suite and therefore may not be feasible in acute settings. Eastman et al. reported a significant reduction in the average time to diagnosis, from 31.2 h to 2.65 h, after transitioning from DSA to CTA for screening and diagnosis of BCVI [19]. After initiation of the CTA program, stroke rate was also reduced from 15.2 to 3.8% as a result of earlier diagnosis and prompt initiation of treatment [15]. In assessment of cost effectiveness, Malhotra et al. reported CTA to be superior to DSA due to its lower cost and low complication risks in evaluation for BCVI in trauma patients [13]. Currently, DSA is mostly reserved as a complementary or confirmatory tool in patients with unclear CTA findings, unexplained neurological deficits or when subsequent intervention has been planned [11,13,18,19].

Magnetic resonance (MR) imaging has the benefits of not requiring ionizing radiation or nephrotoxic contrast administration, and can additionally better detect acute cerebral infarction compared to CT. However, standard MR angiography (MRA) has typically shown low diagnostic accuracy for BCVI detection compared with DSA and CTA, with sensitivities of 50–75% and specificities of 67% compared to DSA [15,16,19,21]. As a result, in conjunction with its longer acquisition times and lack of widespread availability [2,22], MRA is yet to be recommended as a singular modality for BCVI diagnosis [12,22,23,24,25]. It may have specific roles complementary to CTA in certain settings, for example in the differentiation of intramural hematoma from atherosclerotic plaques or intraluminal thrombus, or in follow up of known cases [26,27,28]. More recently, high-resolution MR vessel wall imaging has been studied, and this may have a role in indeterminate cases when performed with luminal imaging.

Duplex ultrasound is rarely used in BCVI diagnosis due to its low sensitivity/specificity and operator dependency. It has limited ability to visualize injuries close to osseous structures or fragments, including injuries involving vessels at the skull base [14,22,29], and minor injuries which may not be associated with disrupted flow.

## 3. Imaging Findings of Arterial Injury

**Figure 1:**
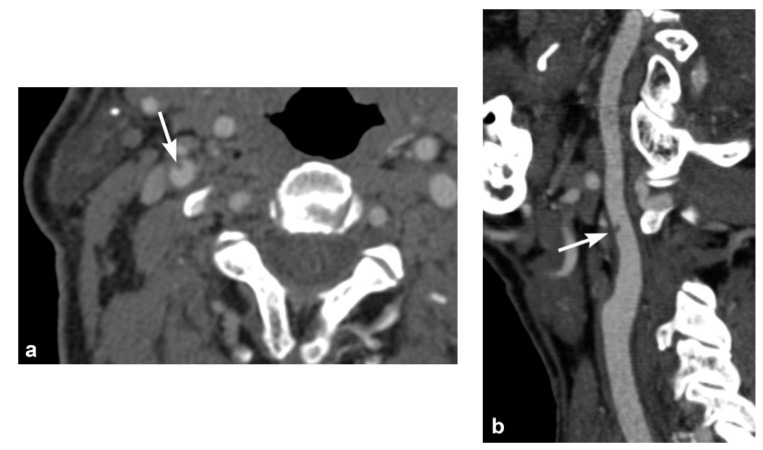
Right internal carotid artery (ICA) injury in a 73-year-old male involved in a motor vehicle accident. (**a**) Axial and (**b**) sagittal oblique multiplanar reformatted multidetector CT angiographic images show a small raised intimal flap in the proximal right ICA.

**Figure 2:**
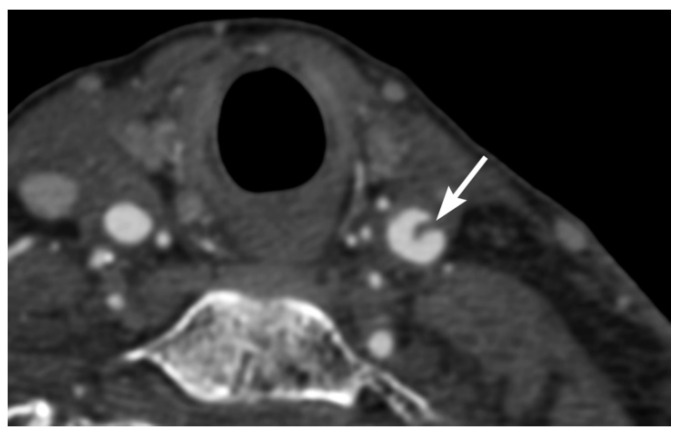
72-year-old male involved in a motor vehicle accident. Axial multidetector CT angiographic image shows a thin linear raised intimal flap in the left common carotid artery.

Imaging findings in BCVI are diverse, paralleling the range of pathological injury which can extend from intimal injury, to complete wall disruption and transection or arteriovenous fistula. Minimal intimal injuries are classified as areas of minor luminal irregularity or mild arterial wall thickening, not typically causing luminal stenosis (<25% stenosis). They can be easily misinterpreted as arterial vasospasm, and therefore a strong index of suspicion may be required. An intimal flap can result from tears with displacement of the intima, which are visualized as linear filling defects extending from the arterial wall [30,31] (Figure 1 and Figure 2). Platelet activation and aggregation is common following intimal disruption, which can lead to more focal thrombus formation at the site of the injury [29,31], and cause further luminal stenosis/occlusion or resultant downstream embolism.

**Figure 3:**
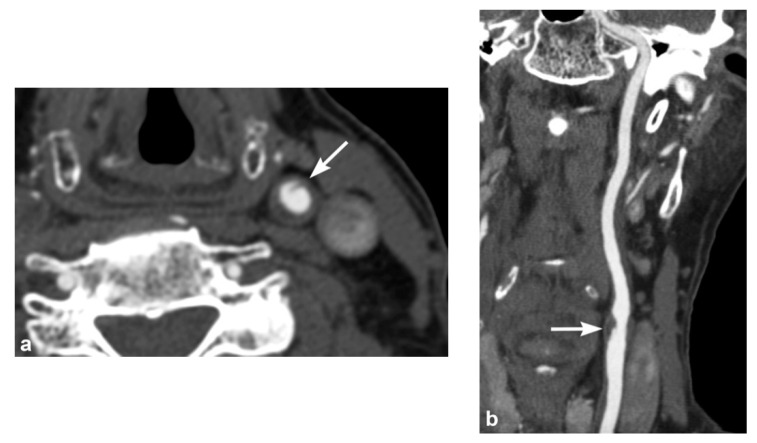
BCVI in a 71-year-old male driver of a motor vehicle accident. (**a**) Axial multidetector CT angiographic image shows a raised intimal flap in the distal left common carotid artery with focal circumferential wall thickening representing blood dissected into the arterial wall. (**b**) Coronal multiplanar reformatted CT angiographic images show blood in the arterial wall at the site of injury, leading to focal luminal narrowing without occlusion.

**Figure 4:**
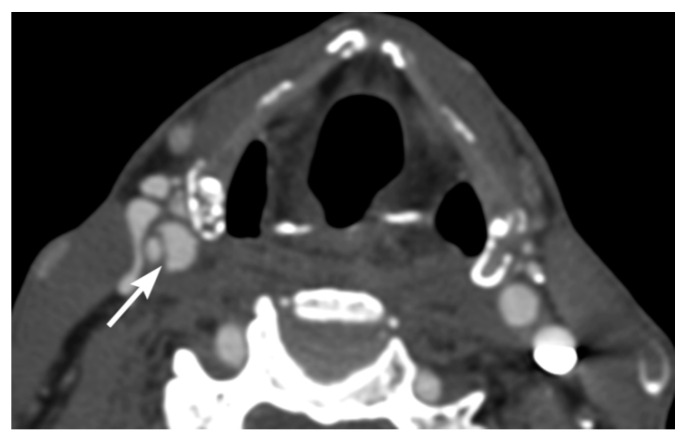
Right common carotid artery injury in a 71-year-old male who presented following a motor vehicle accident. Axial multidetector CT angiographic image shows dissection in the distal right common carotid artery. The located true lumen is moderately narrowed by the false lumen but remains patent.

**Figure 5:**
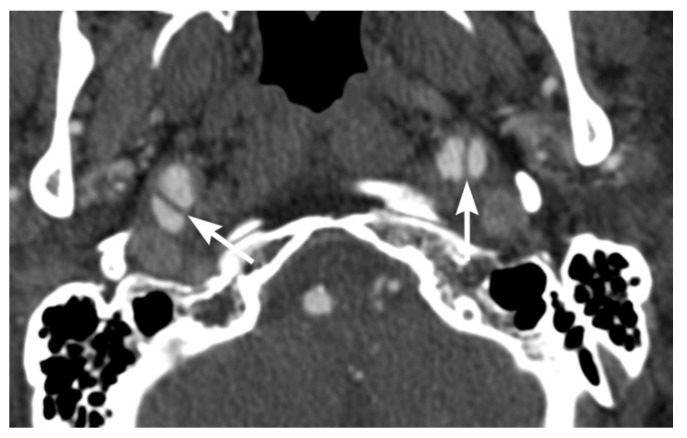
68-year-old male with bilateral BCVI and major thoracic injuries following a motor vehicle accident. Axial multidetector CT angiographic image shows bilateral distal cervical internal carotid artery dissection.

**Figure 6:**
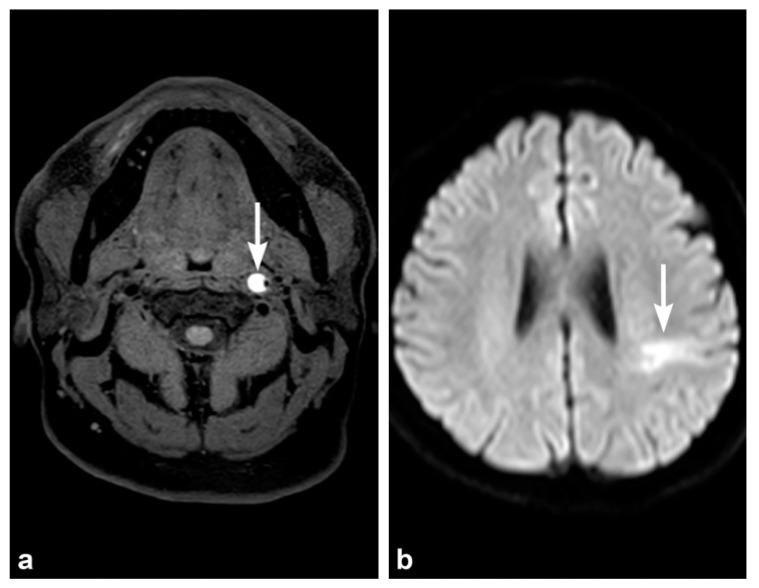
49-year-old male with left internal carotid injury and ischemic stroke (**a**) Axial T1-weighted magnetic resonance (MR) image shows crescentic T1 hyperintensity in the proximal left internal carotid artery compatible with acute intramural hematoma and dissection. There is increased external diameter of the left ICA with severe eccentric luminal narrowing. (**b**) Diffusion-weighted magnetic resonance (MR) image obtained the two days afterwards shows left middle cerebral artery territory stroke. The patient had developed right-sided weakness.

**Figure 7:**
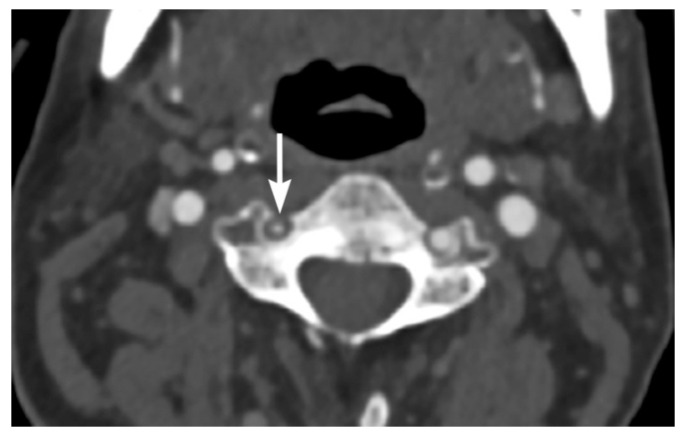
40-year-old female with right vertebral artery intramural hematoma. Axial image multidetector CT angiographic image shows right vertebral artery mural thickening consistent with intramural hematoma with moderate luminal narrowing.

**Figure 8:**
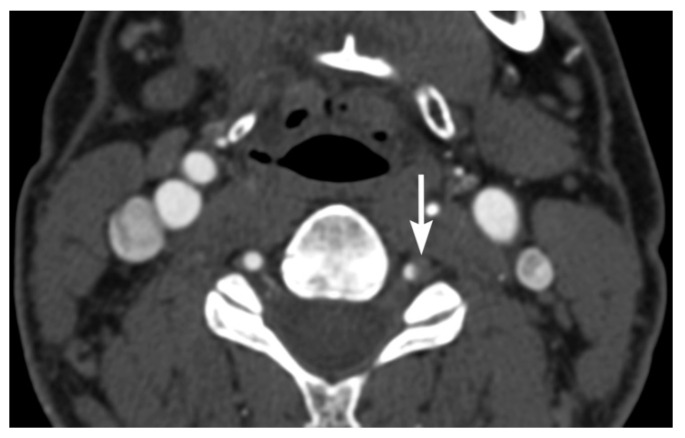
42-year-old male with left vertebral artery injury. Axial image multidetector CT angiographic image shows eccentric left vertebral artery intramural hematoma causing moderate eccentric narrowing of the arterial lumen.

**Figure 9:**
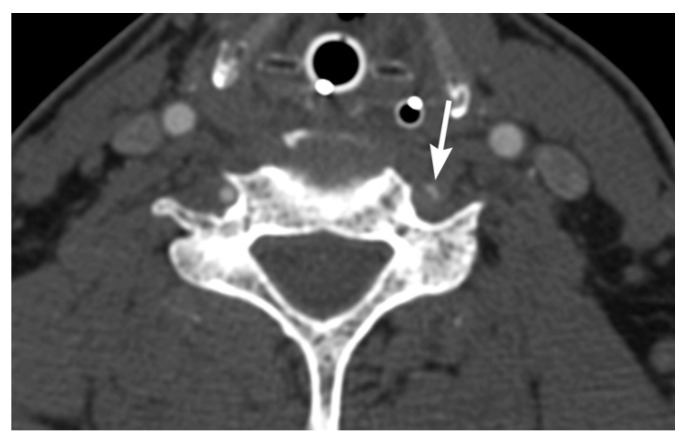
59-year-old male with left vertebral artery injury. Axial multidetector CT angiographic image shows severe eccentric crescentic left vertebral artery narrowing from dissection.

**Figure 10:**
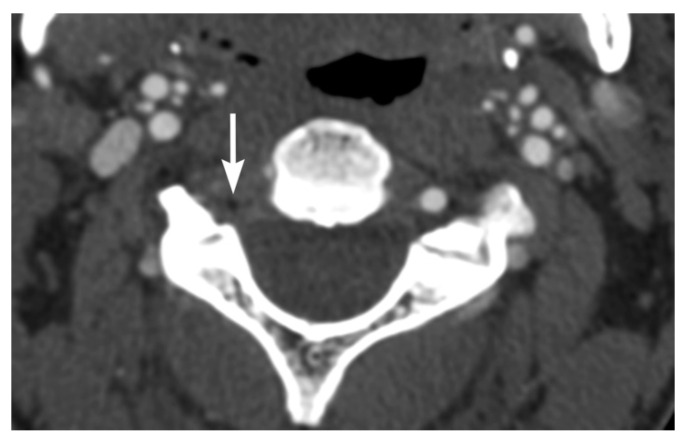
43-year-old male who presented following motor vehicle accident with multiple vertebral fractures and right vertebral artery injury. Axial multidetector CT angiographic image shows occlusion of the right vertebral artery.

**Figure 11:**
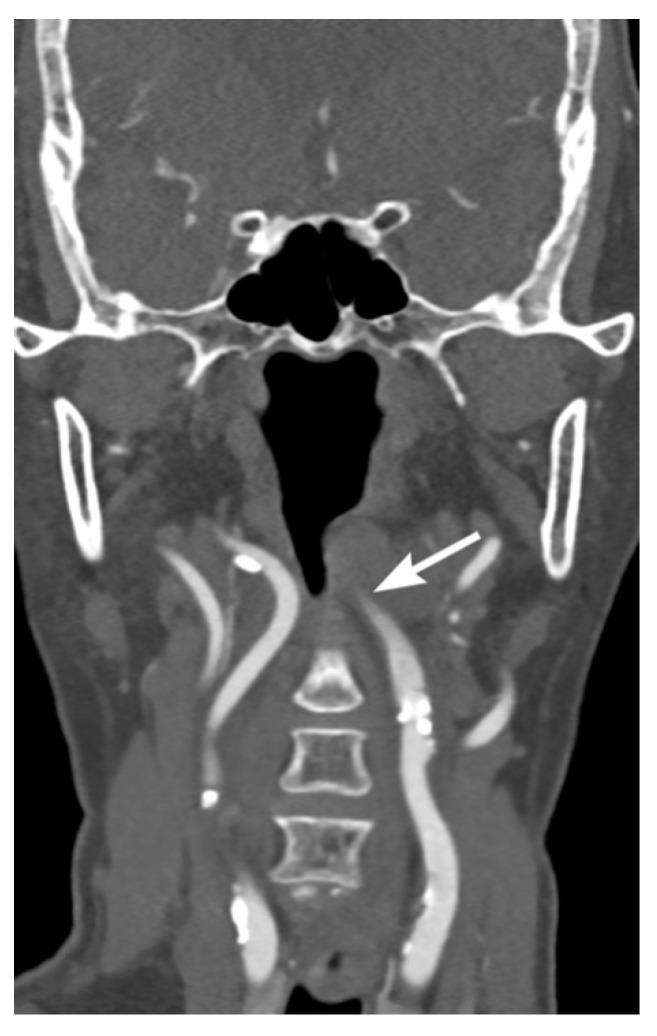
Long segment left internal artery occlusion in a 70-year-old male patient brought in by ambulance with multiple injuries following a motor vehicle accident. Coronal multidetector CT angiographic image shows tapering occlusion of the left internal carotid artery.

**Figure 12:**
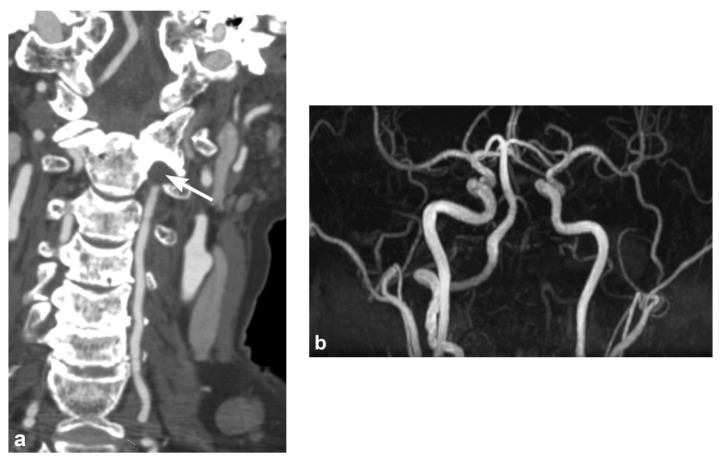
Left vertebral artery occlusion in a 77-year-old male patient involved in a motor vehicle accident. (**a**) Coronal multidetector CT angiographic image shows occlusion of the left vertebral artery with abrupt cut off. (**b**) Three-Dimensional Time-of-Flight MR Angiography of the Circle of Willis shows the long segment left vertebral artery occlusion extended to the intracranial segments.

Intravascular blood can dissect into the vessel wall from intimal injuries (Figure 3, Figure 4 and Figure 5). On the other hand, in adventitiomedial and adventitial injuries, the intima remains intact and intramural hematoma can form within the vessel wall from injury to the vasa vasorum (Figure 6). These injuries can also result in luminal stenosis (Figure 7, Figure 8 and Figure 9) and/or occlusion (Figure 10). In the cervical carotid arteries, radiological appearances of affected vessels on CTA often show tapering before a complete occlusion (Figure 11). In contrast, vertebral artery occlusions tend to more commonly show abrupt arterial cutoffs [27] (Figure 12). The risk of stroke following vessel occlusion is partly dependent on the degree of collateral flow.

**Figure 13:**
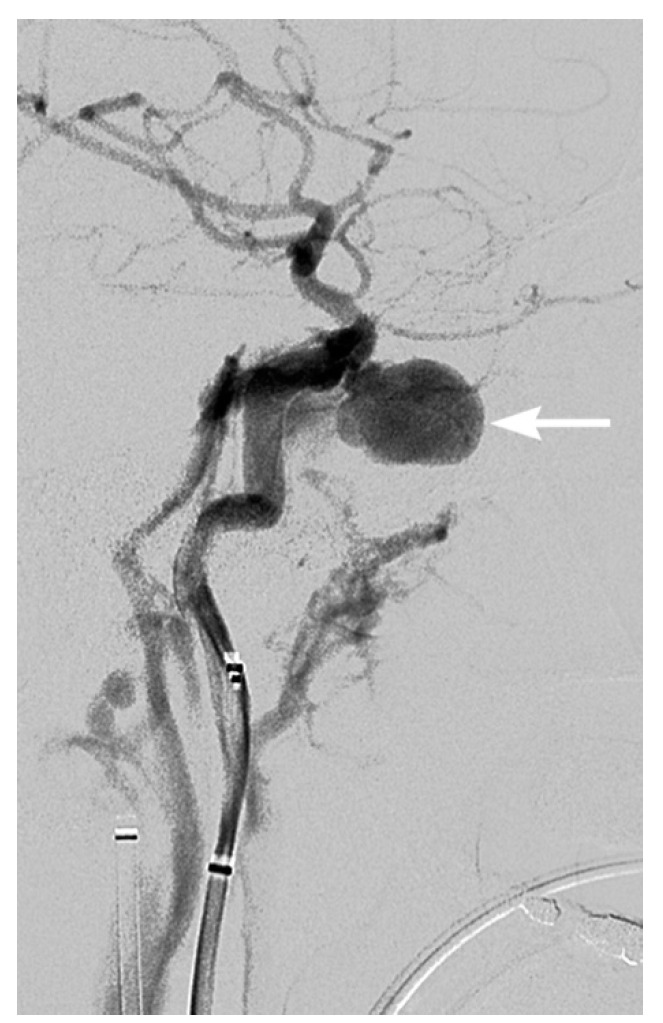
40-year-old male with suicide attempt and fall from height sustaining severe facial and skull base fractures. Diagnostic angiogram showed a large pseudoaneurysm arising from the cavernous left internal carotid artery (arrow) and carotid-cavernous fistula.

Traumatic pseudoaneurysms are variable in appearance and range from focal dilatations to eccentric, saccular outpouchings (Figure 13). An arteriovenous fistula (AVF) may occur when an abnormal communication forms between an artery and an adjacent venous channel. Characteristically, early venous filling during the arterial phase in AVFs may be difficult to detect with single-phase CT angiography. Other features such as dilated draining veins may provide a clue to the diagnosis, and time resolved MR angiography may also be helpful [32]. Arterial transection with free extravasation of contrast represents the most severe form of BCVI. Contrast pools from the injured vessel with typically minimal arterial filling downstream from the site of injury.

The Denver scale described by Biffl et al. is the one of the most commonly used grading scales for classification and clinical prognostication [29]. Increasing grades on the Denver scale correspond to more severe vascular injuries: grade I injury represents vessel wall irregularity, dissection, or intramural hematoma with <25% luminal stenosis; grade II injury includes dissection or intramural hematoma with >25% luminal stenosis; grade III injury corresponds to a pseudoaneurysm; grade IV injury represents complete vessel occlusion; and grade V injury refers to vessel transection with active extravasation or an arteriovenous fistula [29]. For carotid artery injuries, stroke rates increase with increasing injury grade. In vertebral artery injuries, stroke incidence and neurologic outcome have been shown to not directly correlate with injury grade. Various grades can range over injuries which may occur individually or across varying combinations. A study of 171 patients with 236 blunt cerebrovascular injuries showed the relative distribution of injury occurrence as: grade I (58%), grade II (22%), grade III (14%), grade IV (1%), and grade V (3%) [2].

## 4. Screening for Blunt Cerebrovascular Injury

As there may be a latent period from injury to onset of symptoms, with up to 80% of patients with BCVI initially asymptomatic, effective imaging screening is important for timely diagnosis of BCVI [4,8]. Nevertheless, it would be unjustified to perform imaging in all trauma patients to screen for BCVI. Therefore, appropriate pre-imaging screening criteria are required to identify the correct high-risk population for screening and screen out those at low risk for BCVI to minimize unnecessary imaging. Current screening protocols are based on both patient signs and symptoms, and clinical and radiological risk factors from their injury mechanisms and injury patterns.

The two most widely used screening protocols developed by Miller et al. [33] in Memphis and Biffl et al. [34,35] in Denver, include signs such as arterial hemorrhage, cervical bruit, expanding cervical hematoma, focal neurologic deficit, neurologic examination incongruous with head CT findings, or stroke on head CT; and high energy transfer mechanisms with additional injuries such as LeFort II or III facial fractures, basilar skull fractures with carotid canal involvement, complex skull fractures, cervical-spine fractures or subluxation, diffuse axonal injury with GCS <6, near-hanging with anoxic brain injury, thoracic injuries, or blunt cardiac rupture. These criteria have formed the basis of guidelines published by the Western Trauma Association (WTA) in 2009 [36] and the Eastern Association for the Surgery of Trauma (EAST) in 2010 [12]. The recommendations from WTA are based on published observational studies and expert opinion of Western Trauma Association members. The EAST recommendations are level II and II recommendations based on a systematic review from 1965 to 2005, which recommend diagnostic evaluation in patients defined by the criteria for BCVI screening (level II and level II recommendations supported by prospective and retrospective observational studies or uncontrolled retrospective studies). Latest recommendations from the EAST [37] also suggest benefit for patients undergoing standardized screening with a low potential for harm.

More recent studies have proposed expanding the criteria for screening to include clinical/radiological risk factors such as thoracic injuries, scalp degloving, and thoracic vascular injuries [35,38,39]. Screening with more liberal criteria would result in an increased number of BCVI detected, but will also increase the percentage of the trauma population screened. Nonetheless, despite the use of extensive risk factors for screening, several groups using whole body multi-slice screening CTA have suggested that up to 30% of patients with BCVI have no risks factors in which the diagnosis may still be overlooked [18].

## 5. Treatment and Follow-Up

**Figure 14:**
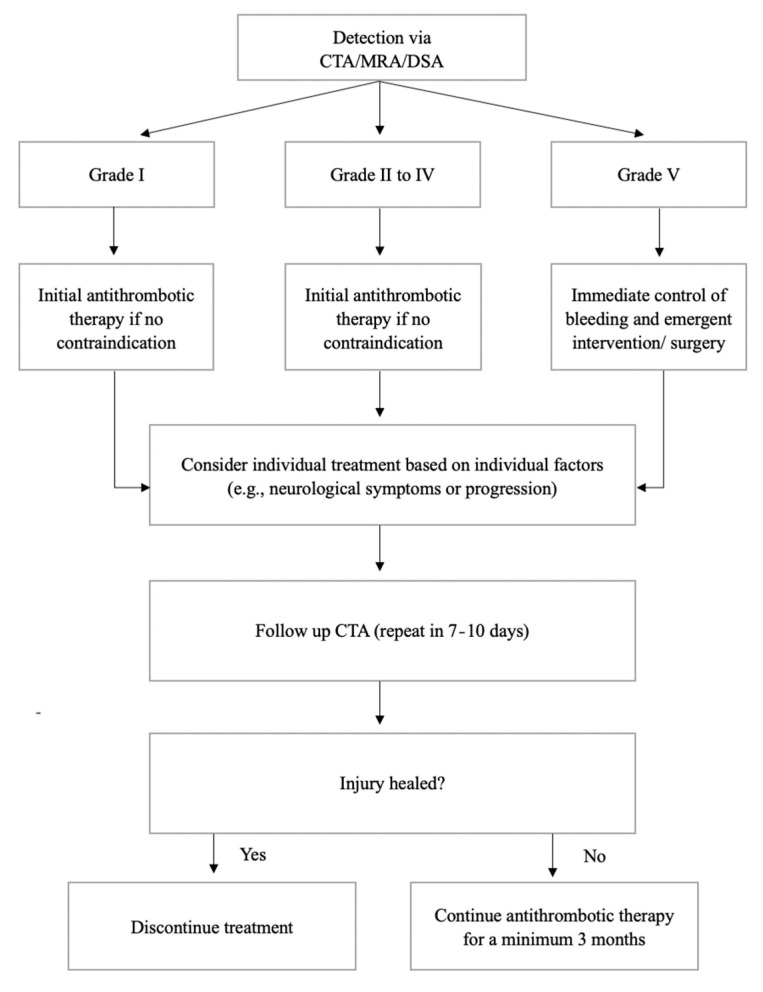
Treatment algorithm based on grade of BCVI.

Treatment strategies include antithrombotic therapy, endovascular intervention, and surgical repair. Given the potential for high morbidity and mortality, expectant management for BCVI is not usually considered unless there are contraindications to treatment [40]. There is evidence for reduced neurologic morbidity and mortality in treated BCVI patients, and indirectly better outcomes following treatment are also seen in the literature of penetrating cerebrovascular injuries and non-traumatic cerebrovascular dissections. Antithrombotic therapy should be initially commenced in all patients if there is no contraindication (e.g., active bleeding) [14]. Low molecular weight heparin is recommended [14]. Antiplatelet agents (clopidogrel or aspirin) may show similar efficacy for stroke prevention and are alternatives if the patient has contraindications for heparin [40,41,42,43]. The use of dual antiplatelets has not been shown to be more effective, and may increase the risk of bleeding. Clinical recommendations for endovascular treatment vary. Grade I injuries typically only require antithrombotic therapy, but other injures may require consideration of endovascular treatment [22]. In practice, individual factors such as site of injury, injury progression, patient neurological symptoms, and local expertise (complication rates, stent patency and stroke rates) tailor recommendations for endovascular treatment. In grade V injuries with active bleeding, guidelines recommend immediate attempts to control bleeding, and emergent intervention/surgery [12] (Figure 14).

After initial management, an early follow up CTA in 7–10 days is helpful as many cases may be normal and treatment may then be halted [14,44]. After this initial period, follow-up studies indicate that healing of the vessel is inversely associated with initial injury grade [45]. However, the majority of BCVIs, up to 84%, may show improvement or complete resolution over time [46]. Of injuries that may improve or resolve, a prior study reported that 76% of changes occur within 1 month of the initial injury, and 24% occur by 3 months [46]. Beyond 3 months, no additional improvement or resolution generally occurs [46]. Short term follow-up CTA within 3 months may have the highest diagnostic yield in assessing for injury change, which may also determine whether continuation or cessation of antithrombotic treatment is required following discharge [14].

## 6. Conclusions

BCVI is an often silent injury in trauma patients with a potential for ischemic stroke. Imaging plays a major role in diagnosis and CTA is the most common modality for initial screening and diagnosis. Vessel wall intimal injuries, intraluminal thrombus, dissection, intramural hematoma, pseudoaneurysm, vessel transection, and arteriovenous fistula, are potential findings to be considered in approach to imaging. A risk stratification strategy based on clinical and radiological risk factors from the injury mechanisms/pattern and patient symptoms can determine which trauma patients are most at risk of BCVI and subsequently can benefit from targeted screening.

## Data Availability

Not applicable.
